# Magnesium Bisglycinate Supplementation in Healthy Adults Reporting Poor Sleep: A Randomized, Placebo-Controlled Trial

**DOI:** 10.2147/NSS.S524348

**Published:** 2025-08-30

**Authors:** Julius Schuster, Igor Cycelskij, Adrian Lopresti, Andreas Hahn

**Affiliations:** 1 Institute of Food and One Health, Leibniz University Hannover, Hanover, Lower Saxony, Germany; 2 College of Science, Health, Engineering and Education, Murdoch University, Perth, Australia

**Keywords:** insomnia, sleep quality, magnesium bisglycinate, nutritional supplementation, randomized controlled trial

## Abstract

**Purpose:**

To assess the effects of magnesium bisglycinate supplementation on insomnia symptoms in healthy adults reporting poor sleep quality.

**Patients and Methods:**

This randomized, double-blind, placebo-controlled trial enrolled 155 adults aged 18–65 years with self-reported poor sleep quality. Participants were randomly assigned to either magnesium bisglycinate supplementation (250 mg elemental magnesium, daily) or placebo capsules. Sleep quality was assessed using the Insomnia Severity Index (ISI) and additional psychological questionnaires at baseline and multiple time points throughout the study. Generalized linear mixed models (GLMM) adjusted for baseline ISI scores, age, sex, body mass index, and occupation were applied.

**Results:**

The magnesium bisglycinate group showed a significantly greater reduction in ISI scores compared to the placebo group from baseline to Week 4 (−3.9 [95% CI: −5.8 to −2.0] vs −2.3 [95% CI: −4.1 to −0.4], respectively; p = 0.049). The effect size was small (Cohen’s d = 0.2), indicating a modest benefit. Exploratory analyses suggested notably greater improvements among participants reporting lower baseline dietary magnesium intake, potentially indicating a subgroup of high responders. No significant differences were observed in other psychological outcomes.

**Conclusion:**

Magnesium bisglycinate supplementation modestly improved insomnia severity in adults reporting poor sleep quality. Future research should include objective sleep assessments, longer intervention periods, and better characterization of potential high responders by systematically assessing baseline dietary magnesium intake and status.

**Clinical Trial Registration Name:**

Effect of magnesium bisglycinate supplementation on sleep and fatigue parameters in healthy adults reporting poor sleep quality; https://drks.de/search/en/trial/DRKS00031494 DRKS-ID: DRKS00031494.

## Introduction

Sleep disturbance and insomnia symptoms are very prevalent in modern society, often disrupting regular sleep patterns and overall well-being.^1^,^2^ Approximately one-third of the global population reports dissatisfaction with their sleep, with 6–15% meeting the clinical criteria for insomnia disorders.^3^ Insomnia symptoms were reported twice as often in women, consistent with global data on gender differences in sleep disturbances.^4^ While restorative sleep can mitigate some of the effects of short-term sleep deprivation,^5^ persistent sleep disruption is associated with cognitive impairment,^6^ mood disturbance,^7^ and other mental health problems.^8^ It is also associated with broader health risks, including cardiovascular disease and, in men, higher mortality rates.^9^,^10^

Cognitive Behavioral Therapy for Insomnia (CBT-I) is considered the most effective treatment for chronic insomnia.^11^ It improves sleep efficiency by 10–20% and reduces insomnia severity in up to 60% of patients, with benefits that often last long-term.^11^ For severe insomnia, benzodiazepines and non-benzodiazepine hypnotics (eg, zolpidem, zaleplon) can provide short-term relief but pose risks such as dependence, tolerance and next-day cognitive or motor impairment, especially with prolonged use.^12^,^13^ Over-the-counter options like antihistamines (eg, diphenhydramine) are more accessible for occasional sleep disturbances, but rapid tolerance and next-day sedation limit their daily use in people reporting only mild insomnia.^14^ While melatonin is a widely used dietary supplement for sleep regulation, its long-term efficacy and safety remain in question.^15^,^16^ Consequently, there is growing interest in alternative nutritional interventions—magnesium supplementation has emerged as another promising option.

Magnesium is essential for cardiovascular, skeletal, and neural function, acting as a cofactor in numerous enzymatic processes that support cellular and metabolic balance.^17^ Suboptimal magnesium levels are common and have been linked to various chronic conditions, yet they often go undetected due to imprecise diagnostic criteria.^18^ Magnesium plays a role in sleep regulation, with both observational and some clinical studies linking higher magnesium intake and magnesium supplementation to improved sleep outcomes. Observational research associates greater magnesium consumption with better sleep quality, including shorter sleep onset latency, longer sleep duration, and reduced daytime sleepiness.^19^ Clinical trials further suggest that magnesium supplementation enhances sleep efficiency and reduces insomnia severity, potentially through mechanisms such as increased melatonin production and reduced cortisol levels.^20^,^21^ A recent systematic review found an association between magnesium status and sleep quality in observational studies, but highlighted inconsistencies in interventional trials: two RCTs showed improvements in sleep efficiency, time, or latency, while three found no significant effects.^22^ Given that these trials included only 247 participants in total, there is a clear need for well-powered studies with larger sample sizes.

Magnesium bisglycinate, a chelated compound combining magnesium with glycine, has not been specifically studied for its impact on sleep. Existing research is limited to applications like heart rate variability and pregnancy-induced leg cramps, where some benefits have been observed.^23^,^24^ While some studies suggest organic magnesium salts may have slightly higher bioavailability than inorganic forms, clinical evidence directly comparing their absorption remains limited.^25^

Although the glycine content in magnesium bisglycinate is relatively lower (approximately 1.54 g per 250 mg of elemental magnesium), its co-presence may produce synergistic effects with magnesium in supporting sleep physiology. Glycine has also been explored for its sleep-promoting properties due to its ability to interact with key neurotransmitter systems, including the N-methyl-D-aspartate (NMDA) receptors.^26^ While evidence remains inconclusive, some studies suggest that glycine supplementation at a dose of 3 g can enhance sleep quality and reduce daytime fatigue, highlighting its potential as a supportive agent in sleep management.^27^,^28^

This four-week, placebo-controlled study was conducted as a large, nationwide trial across Germany. It aimed to provide real-world evidence through home-based assessments and to offer new insights into the potential benefits of magnesium bisglycinate for individuals reporting poor sleep.

## Materials and Methods

### Trial Design and Participants

The study was a two-arm, parallel-group, four-week, double-blind, placebo-controlled trial conducted in accordance with CONSORT guidelines. It was approved by the Ethics Committee of the Medical Association of Lower Saxony (Hannover, Germany) and designed in accordance with the principles of Good Clinical Practice (GCP) and the Declaration of Helsinki. This trial was registered at the German Clinical Trials Register (DRKS) (DRKS00031494) at the time of recruitment.

Participants were recruited across Germany through various advertisements between March and May 2023. Initial enrollment began on April 20, 2023. A second wave followed in May to reach the target sample size. Recruitment was conducted online, and interested participants were directed to a screening questionnaire to assess eligibility. This online questionnaire consisted of the Regensburg Insomnia Scale (RIS)^29^ and a short survey to collect some general background information to help assess participant eligibility criteria. Individuals who passed this initial screening were subsequently invited to participate in the study and provided detailed information about the study objectives, procedures, potential risks and benefits, and data confidentiality. Informed consent was obtained electronically from each participant before enrollment.

Eligible participants were adults between the ages of 18 and 65 who self-reported being in good health, with no history of chronic disease or conditions requiring ongoing treatment. Health status was assessed using a pre-screening questionnaire that excluded individuals with known chronic illnesses or conditions requiring ongoing treatment. Other inclusion criteria included poor sleep for more than four weeks, as assessed by a RIS score greater than 12, and a body mass index between 18.5 and 35 kg/m². Individuals with treated thyroid disorders, such as hyperthyroidism, hypothyroidism, and Hashimoto’s disease, were eligible to participate as long as there had been no change in their treatment regimen within the previous three months. In addition, all participants were required to refrain from taking any dietary supplements for two weeks prior to the start of the intervention.

Individuals were ineligible if they engaged in rotational-shift work or had a diagnosed sleep disorder (such as sleep apnea) requiring treatment. They were also excluded if they had severe chronic diseases, including cancer, epilepsy, renal or liver insufficiency, rheumatoid arthritis, multiple sclerosis, or gastrointestinal conditions like Crohn’s disease, pancreatic insufficiency or ulcerative colitis. Regular use of medications affecting sleep or mood (eg, antidepressants, beta-blockers, sedatives, antihistamines) or laxatives was not permitted. Exclusion also applied to those consuming >200 mg/day of magnesium or >1 g/day of glycine within one month prior to the study, individuals with alcohol, drug, or medication dependence, those unable to provide consent, pregnant or breastfeeding women, recent clinical trial participants (<30 days), or those with planned surgery within three months. Major sleep rhythm changes for professional or personal reasons were also exclusion criteria.

### Intervention

After randomization, participants received their study packages by post. They were instructed to take two capsules daily, 30–60 minutes before bedtime, while maintaining their usual diet, lifestyle habits, and physical activity throughout the intervention period. The magnesium bisglycinate supplement used in this study was manufactured by Biogena, Salzburg, Austria. Each capsule contained 893 mg of magnesium bisglycinate, corresponding to 125 mg of magnesium and 761.5 mg of glycine, providing a total daily dose of 250 mg of magnesium and 1523 mg of glycine. The placebo capsules contained cellulose and were visually identical to the test product. The capsule shells were made of hydroxypropyl methyl cellulose.

Adherence was monitored via questionnaires and self-reported capsule counts. Individuals in the placebo group were offered the active magnesium bisglycinate supplement after unblinding.

### Endpoints

Participants completed online questionnaires at baseline (Day 0), weekly throughout the four-week intervention (Days 7, 14, 21), and at the end of the study (Day 28) to assess the primary and secondary outcomes described below (Table 1).

**Table 1:** Endpoints and Assessment days

	Screening	Day 0	Day 7	Day 14	Day 21	Day 28
*Primary endpoint*						
ISI** **		X		X		X
*Secondary endpoints*						
RIS** **	X	X				X
SQS** **		X	X	X	X	X
PANAS** **		X	X	X	X	X
FSS** **		X	X	X	X	X
ESS** **		X	X	X	X	X
PSS** **		X				X
PHQ-4** **		X		X		X

**Abbreviations**: ISI, Insomnia Severity Index; RIS, Regensburg Insomnia Scale; SQS, Single-Item Sleep Quality Scale; PANAS, Positive and Negative Affect Schedule; FSS, Fatigue Severity Scale; ESS, Epworth Sleepiness Scale; PSS, Perceived Stress Scale; PHQ-4, Patient Health Questionnaire-4.

#### Primary Endpoint

The Insomnia Severity Index (ISI), developed and validated by Bastien et al,^30^ is a widely recognized tool for assessing insomnia symptoms in research settings, with a validated German version available for research use.^31^ It evaluates both nighttime and daytime aspects of insomnia through seven items assessing sleep difficulties, satisfaction with sleep, functional impairment, and distress. As per the study protocol, the ISI was administered biweekly. The ISI was used under a non-commercial academic license from MAPI Research Trust for non-funded academic research purposes.^32^

#### Secondary endpoints

The Regensburg Insomnia Scale (RIS) was used for initial screening and as a secondary outcome measure at screening, baseline, and day 28.^29^ The Sleep Quality Scale (SQS), a single-item measure (0–10), assessed sleep quality on a weekly basis.^33^ Fatigue was evaluated using the Fatigue Severity Scale (FSS), which includes nine items assessing physical and cognitive fatigue on a 7-point Likert scale.^34^,^35^ The Epworth Sleepiness Scale (ESS) assessed daytime sleepiness likelihood.^36^ The Positive and Negative Affect Schedule (PANAS) measured emotional states, with separate 10-item scales for positive and negative affect.^37^ The Perceived Stress Scale (PSS) evaluated perceived stress over the past month,^38^ while the Patient Health Questionnaire-4 (PHQ-4) screened for symptoms of depression and anxiety.^39^,^40^

RIS,^29^ SQS,^33^ and FSS^34^ are freely available for non-funded academic research by the authors. ESS,^41^ PANAS^42^ and PSS^43^ are available for non-funded academic research via the MAPI Research Trust. The PHQ-4 is available without copyright restrictions by Pfizer.^44^

In addition to these outcome measures, an exploratory analysis assessed the intake of magnesium-rich foods and mineral water using a brief, non-validated dietary rating scale developed specifically for this study. This scale categorized participants based on their self-reported frequency of consuming magnesium-rich foods. While it provided general insights into dietary habits, it was not validated against established dietary assessment methods.

### Sample Size and Randomization

A priori power analysis was performed to estimate the necessary sample size. Using the ISI as an outcome measure, an effect size (Cohen’s d) of 0.69 was found in a study examining the effects of magnesium supplementation on sleep quality in older people.^20^ As this study targeted a broader age range and multiple outcome measures were utilized, a more conservative effect size of 0.45 was used for the sample size calculation. Assuming 80% power and a type 1 error rate (α) of 5%, the number of participants required per group to detect an effect on the ISI was estimated as 62. Considering a 15% drop-out rate, the goal was to have a minimum of 75 participants in each group.

All eligible participants enrolled in the study were randomly assigned in a 1:1 ratio to one of two groups (magnesium bisglycinate or placebo) under double-blind conditions. The random allocation sequence was generated by an independent investigator who was not involved in recruitment, data collection, or adjudication. Randomization was stratified by RIS score, sex, and age (in descending order) using a custom block randomization procedure. The sequence was generated using a computerized block randomization method and stored in a secure, access-restricted database accessible only to the independent investigator overseeing randomization. Participants were enrolled by the study team, who were blinded to group allocation. After enrollment, an independent investigator assigned participants to their respective intervention groups.

Allocation concealment was maintained by ensuring that neither participants, investigators, outcome assessors, nor data analysts had access to the randomization sequence or group assignments until all outcome data were collected. A blinded review and initial analysis were conducted before unblinding to ensure objective data interpretation.

### Statistical Methods

Descriptive statistics for quantitative variables, such as BMI, age, and weight, were presented as mean ± standard deviation (SD). To evaluate the normality of these variables, the Shapiro–Wilk test was conducted prior to further statistical comparisons. For baseline comparisons between groups, independent samples t-tests were utilized for normally distributed continuous variables, while chi-squared tests were applied to compare categorical variables.

Primary outcome assessments followed the intention-to-treat (ITT) principle, ensuring all randomized participants were included in the analysis based on their originally assigned group, regardless of adherence to the study protocol. A supplementary per-protocol (PP) analysis was conducted, including participants who met the predefined adherence criteria—consuming at least 80% of the capsules, missing no more than 7 days of the 28-day study period, and completing the trial while following other protocol requirements.

To assess differences between the intervention groups for both primary and secondary outcomes, Generalized Linear Mixed Models (GLMMs) were employed to analyze changes in mean scores from baseline to day 28. Fixed effects in the GLMM analyses included baseline scores and potential confounding variables such as age, BMI, sex, and occupation. All statistical analyses were performed using SPSS software (IBM SPSS Statistics, version 28.0.1.0; Chicago, IL, USA).

To examine potential associations between dietary magnesium intake and changes in ISI scores, a correlation analysis was conducted using Spearman’s rank correlation coefficient. Given the non-validated nature of the dietary assessment tool and its ordinal 5-point scale, this analysis was considered exploratory. The correlation was computed using Python (version 3.10.12) and the SciPy library. Statistical significance for all tests was set at p < 0.05.

In addition to the primary analyses using GLMM, independent samples t-tests were conducted for all primary and secondary outcomes at Day 28 to provide direct cross-sectional between-group comparisons at the study endpoint. These comparisons, presented in Table 2, serve as additional sensitivity analyses complementing the longitudinal results.

**Table 2:** Changes in Outcome Measures From Baseline to week 4 (Intention-to-Treat Analysis)

	Endpoint comparison			Adjusted mean change from baseline after 4 weeks				
	Placebo n = 76 Mean ± SD	Magnesium bisglycinate n = 77 Mean ± SD	p-value (*t*-test)	Placebo n = 76 Mean (95% CI)	Magnesium bisglycinate n = 77 Mean (95% CI)	Difference between groups (95% CI)	p-value (GLMM)	Cohen’s d
***Primary endpoint***								
**ISI**	12.8 ± 5.3	11.0 ± 4.8	0.037*	−2.3 (−4.1 to −0.4)	−3.9 (−5.8 to −2.0)	1.6 (0.0 to 3.3)	0.049*	0.20
***Secondary endpoints***								
**RIS**	17.2 ± 4.5	15.7 ± 5.3	0.072	−1.1 (−2.7 to 0.5)	−2.0 (−3.7 to −0.4)	1.0 (−0.5 to 2.4)	0.186	0.13
**SQS**	4.6 ± 2.1	5.3 ± 2.2	0.071	0.3 (−0.5 to 1.2)	1.0 (0.2 to 1.9)	−0.7 (−1.4 to 0.1)	0.069	0.18
**ESS**	7.5 ± 4.1	7.6 ± 4.4	0.874	−1.8 (−3.0 to −0.6)	−2.0 (−3.2 to −0.8)	0.3 (−0.8 to 1.3)	0.624	0.05
**FSS**	40.8 ± 11.1	37.9 ± 11.6	0.142	−1.8 (−5.0 to 1.5)	−3.0 (−6.3 to 0.3)	1.3 (−1.6 to 4.2)	0.391	0.09
**PSS**	19.6 ± 7.2	18.0 ± 7.0	0.218	−2.0 (−4.3 to 0.3)	−2.4 (−4.8 to −0.1)	0.4 (−1.7 to 2.5)	0.708	0.04
**PANAS (+)**	28.6 ± 6.8	29.5 ± 7.5	0.456	1.3 (−1.1 to 3.6)	1.0 (−1.4 to 3.3)	0.3 (−1.8 to 2.4)	0.771	0.03
**PANAS (-)**	20.2 ± 7.9	20.1 ± 6.9	0.931	−3.0 (−5.2 to −0.8)	−2.9 (−5.0 to −0.7)	0.1 (−1.8 to 2.0)	0.894	0.01
**PHQ-4 Total**	4.2 ± 2.7	3.9 ± 2.7	0.455	0.1 (−0.6 to 0.8)	−0.0 (−0.8 to 0.7)	−0.2 (−0.8 to 0.5)	0.634	0.05
**PHQ-4 (D)**	2.1 ± 1.4	1.9 ± 1.4	0.326	−0.1 (−0.5 to 0.3)	−0.2 (−0.6 to 0.2)	0.1 (−0.3 to 0.4)	0.711	0.04
**PHQ-4 (A)**	2.1 ± 1.5	2.0 ± 1.5	0.677	0.2 (−0.2 to 0.7)	0.2 (−0.2 to 0.7)	0.0 (−0.4 to 0.4)	0.860	0.02

**Notes**: Mean changes (95% CI) represent the average difference in scores from baseline to week 4, with 95% confidence intervals (CI) indicating the range within which the true mean difference is expected to lie. Difference (95% CI) refers to the comparison between groups, showing the estimated difference in score changes between placebo and magnesium bisglycinate groups. *p*-values indicate the statistical significance of the observed differences, with values less than 0.05 considered statistically significant (bolded in the table). Cohen’s *d* represents the effect size, indicating the magnitude of the observed differences between groups. For ISI, RIS, ESS, FSS, PSS, PANAS (-), and PHQ-4 scores, a negative change indicates improvement. For SQS and PANAS (+) scores, increased scores signify improvement. Results are generated from generalized linear mixed-effects models (GLMM) adjusted for age, sex, body mass index, occupation, and corresponding baseline values (time × group interaction). Unadjusted endpoint comparisons using independent-samples *t*-tests are provided for descriptive purposes. Bolded values and an asterisk (*) indicate statistically significant differences (p < 0.05).

**Abbreviations**: ISI, Insomnia Severity Index; lower scores indicate improved sleep quality. RIS, Regensburg Insomnia Scale; lower scores indicate reduced insomnia symptoms. SQS, Single-Item Sleep Quality Scale; higher scores indicate improved sleep quality. ESS, Epworth Sleepiness Scale; lower scores indicate less daytime sleepiness. FSS, Fatigue Severity Scale; lower scores indicate less fatigue. PSS, Perceived Stress Scale; lower scores indicate lower perceived stress. PANAS (+), Positive and Negative Affect Schedule (Positive Affect); higher scores indicate more positive affect. PANAS (-), Positive and Negative Affect Schedule (Negative Affect); lower scores indicate less negative affect. PHQ-4, Patient Health Questionnaire-4; lower scores indicate fewer symptoms of depression (D) and anxiety (A).

## Results

### Study Population

**Figure 1:**
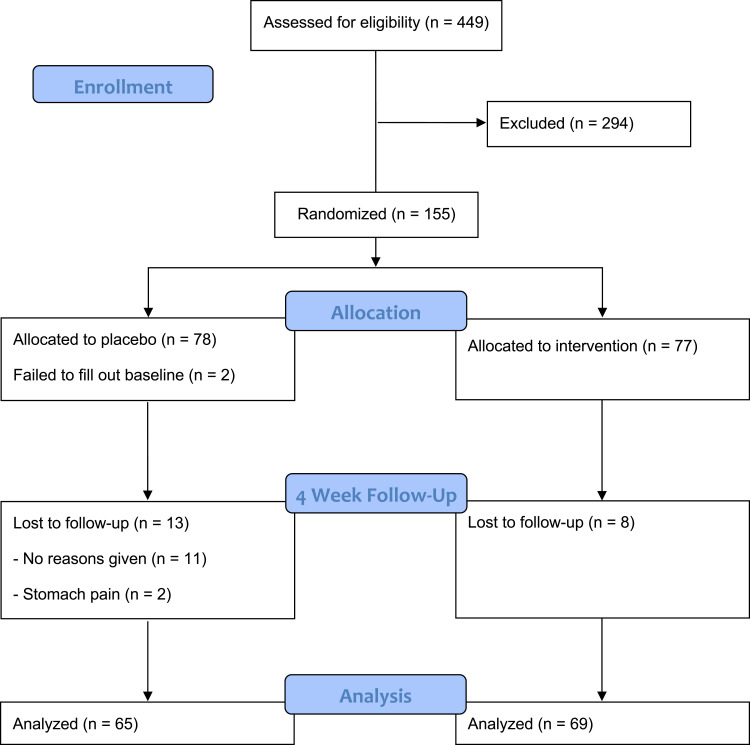
CONSORT flow diagram showing participant progression through enrollment (n = 449), exclusion (n = 294), randomization (n = 155), allocation, follow-up, and analysis.

**Notes**: CONSORT figure adapted from Schulz KF, Altman DG, Moher D. CONSORT 2010 Statement: Updated Guidelines for Reporting Parallel Group Randomised Trials. PLoS Med. 2010;7(3): e1000251. Copyright: © 2010 Schulz et al. Creative Commons Attribution License.

A total of 449 individuals completed the online screening questionnaire, of whom 155 were randomized (Figure 1). Of these, 78 participants were assigned to the placebo group and 77 to the intervention group. Of the randomized participants, 153 completed the baseline survey and were included in the primary outcome analysis on an ITT basis. At the end of the 4-week intervention, 65 participants remained in the placebo group and 69 in the intervention group, resulting in 134 participants completing the study. Two participants in the placebo group withdrew due to stomach pain, while the reasons for withdrawal in other cases (11 in the placebo group and 8 in the intervention group) were unspecified. The trial was conducted as planned and ended upon completion of the second study wave.

Key demographic and clinical characteristics of the participants are presented in Table 3, while baseline scores of all questionnaires are shown in Table 4.

**Table 3:** Baseline Sociodemographic and Clinical Characteristics

Characteristics	Total n = 153	Placebo n = 76	Magnesium bisglycinate n = 77
	Mean ± SD	Mean ± SD	Mean ± SD
**Age**, [years]	41.5 ± 13.1	42.1 ± 12.9	40.9 ± 13.3
**Weight**, [kg]	74.3 ± 16.5	75.5 ± 19.6	73.0 ± 12.7
**BMI**, [kg/m^2^]	25.4 ± 4.8	25.7 ± 5.5	25.1 ± 4.1
**Sex**, n (%)			
Male	31 (20.3)	16 (21.1)	15 (19.5)
Female	122 (79.7)	60 (78.9)	62 (80.5)
**Occupational Status**, n (%)			
Unemployed	6 (3.9)	3 (3.9)	3 (3.9)
Student	33 (21.6)	11 (14.5)	22 (28.6)
Worker	109 (68.0)	59 (73.7)	50 (62.3)
Pensioner	5 (3.3)	3 (3.9)	2 (2.6)
**Physical activity**, n (%)			
Never, rarely (1–2)	52 (34.0)	30 (39.5)	22 (28.6)
Moderate (3–4)	81 (52.9)	32 (42.1)	49 (63.6)
Often (5)	19 (12.4)	13 (17.1)	6 (7.8)
No answer (6)	1 (0.7)	1 (1.3)	0 (0.0)
**Dietary form**, n (%)			
Omnivore	107 (69.9)	53 (69.7)	54 (70.1)
Vegetarian	36 (23.5)	18 (23.7)	18 (23.4)
Vegan	5 (3.3)	3 (3.9)	2 (2.6)
No answer	5 (3.3)	2 (2.6)	3 (3.9)
**Caffeine intake**, n (%)			
None (<1 cup/day)	26 (17.0)	16 (21.1)	10 (13.0)
Moderate	89 (58.2)	43 (56.6)	46 (59.7)
High	38 (38.0)	17 (22.4)	21 (27.3)
**Magnesium-rich diet**, n (%)			
Almost never	23 (15.0)	13 (17.1)	10 (13.0)
Sometimes	39 (25.5)	22 (28.9)	17 (22.1)
Regularly	53 (34.6)	25 (32.9)	28 (36.4)
Often	38 (24.8)	16 (21.1)	22 (28.6)

**Notes**: No statistically significant differences were observed between the groups at baseline except for physical activity *(p=0.039)*.

**Abbreviations**: BMI, body mass index; SD, standard deviation.

**Table 4:** Baseline Scores of Outcome Measures

Characteristics	Total n = 153	Placebo n = 76	Magnesium bisglycinate n = 77
	Mean ± SD	Mean ± SD	Mean ± SD
**ISI**	15.5 ± 3.7	15.7 ± 3.9	15.3 ± 3.5
**RIS**	18.5 ± 4.3	18.7 ± 4.5	18.3 ± 4.2
**SQS**	3.7 ± 1.8	3.6 ± 1.8	3.8 ± 1.9
**ESS**	9.3 ± 4.5	9.5 ± 4.8	9.1 ± 4.2
**FSS**	43.1 ± 10.9	43.8 ± 11.0	42.4 ± 10.9
**PSS**	21.6 ± 7.4	22.2 ± 8.0	21.0 ± 6.7
**PANAS (+)**	27.4 ± 6.7	27.0 ± 6.7	27.8 ± 6.7
**PANAS (-)**	23.1 ± 7.2	23.5 ± 7.6	22.8 ± 6.8
**PHQ-4**	4.7 ± 2.9	5.1 ± 3.1	4.3 ± 2.6

**Abbreviations**: SD, Standard deviation; ISI, Insomnia Severity Index; lower scores indicate improved sleep quality. RIS, Regensburg Insomnia Scale; lower scores indicate reduced insomnia symptoms. SQS, Single-Item Sleep Quality Scale; higher scores indicate improved sleep quality. ESS, Epworth Sleepiness Scale; lower scores indicate less daytime sleepiness. FSS, Fatigue Severity Scale; lower scores indicate less fatigue. PSS, Perceived Stress Scale; lower scores indicate lower perceived stress. PANAS (+), Positive and Negative Affect Schedule (Positive Affect); higher scores indicate more positive affect. PANAS (-), Positive and Negative Affect Schedule (Negative Affect); lower scores indicate less negative affect. PHQ-4, Patient Health Questionnaire-4; lower scores indicate fewer symptoms of depression and anxiety.

### Primary Endpoint: Insomnia Severity Index

**Figure 2:**
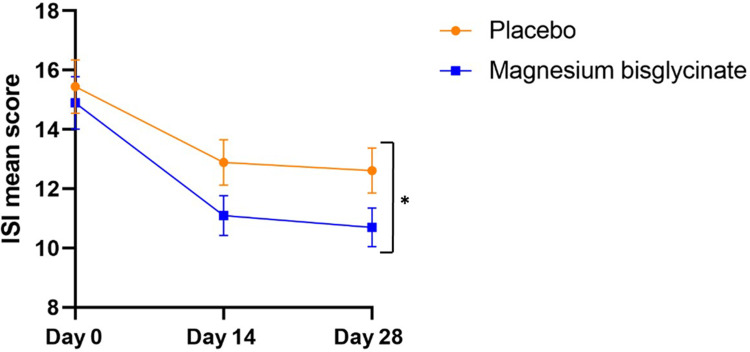
Mean Insomnia Severity Index (ISI) scores over time (intention-to-treat). Data represent estimated means from generalized linear mixed-effects models (GLMM), adjusted for age, sex, body mass index, and occupation. Error bars indicate the standard error of the mean (SEM). Asterisks (*) denote statistically significant differences (*p* = 0.049) with a small effect size (*d* = 0.2).

Based on the intention-to-treat GLMM analysis detailed in Table 2, the magnesium bisglycinate group had a statistically significantly greater between-group mean reduction in ISI scores from baseline to Week 4 [−3.9 (95% CI, −5.8 to −2.0)] compared to the placebo group [−2.3 (95% CI, −4.1 to −0.4)] (p = 0.049) (Figure 2). The effect size for this between-group difference, as measured by Cohen’s d, was small (d = 0.2), indicating a modest treatment effect. Figure 2 illustrates the change in ISI scores over the intervention period. Both groups exhibited significant within-group improvements during the first two weeks (data not shown). Within the magnesium bisglycinate group, there was a 28% reduction in ISI scores from baseline to day 28 (p = 0.001), compared to an 18% reduction in the placebo group (p = 0.001). These within-group reductions suggest that both groups experienced improvements in self-reported insomnia severity, but the magnesium bisglycinate group demonstrated a significantly greater benefit over time.

A clinically significant improvement in insomnia was defined as a reduction of 6 points or more in the Insomnia Severity Index (ISI) score according to Yang et al.^45^ In our study, 41 participants (30%) showed such clinically significant changes. Within this group, 15 (11%) were in the placebo group and 26 (19%) in the verum group (data not shown).

In addition to the primary GLMM analysis, an independent samples *t*-test comparing groups at Day 28 confirmed a statistically significant difference in ISI scores between the magnesium bisglycinate and placebo groups (p = 0.037; Table 2).

### Per-Protocol Analysis

The per-protocol (PP) analysis of 134 participants who strictly adhered to the study protocol showed results consistent with the ITT evaluation of ISI scores. From baseline to day 28, the magnesium bisglycinate group had a significantly greater reduction in ISI scores [−5.0 (95% CI, −7.5 to −2.4)] compared to the placebo group [−3.1 (95% CI, −5.5 to −0.8)] (p = 0.035).

### Secondary Endpoints

**Figure 3:**
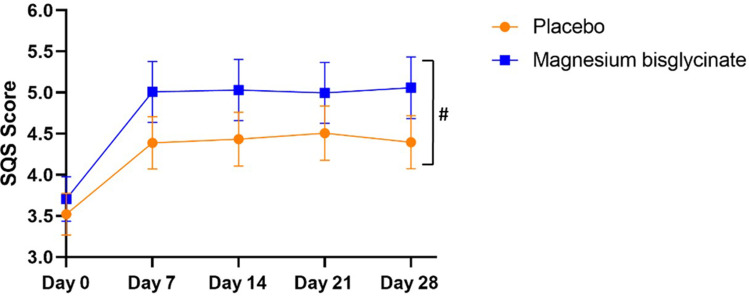
Mean Single-Item Sleep Quality Scale (SQS) scores over time. Data represent estimated means from generalized linear mixed-effects models (GLMM), adjusted for age, sex, body mass index, and occupation. Error bars indicate the standard error of the mean (SEM). (#) indicates a trend toward significance (*p* < 0.10).

No statistically significant time × group interaction was observed in the other questionnaires, as determined by both ITT and PP analyses (Table 2). The Single-Item Sleep Quality Scale mean scores (higher scores indicate better sleep quality) improved significantly in the first week (*p* < 0.001) in both groups, with no significant changes thereafter (Figure 3). Although the magnesium bisglycinate group exhibited greater improvement compared to the placebo group, this difference did not reach statistical significance (*p* = 0.069).

Independent samples t-tests were also conducted for secondary outcomes at Day 28 (Table 2). No statistically significant between-group differences were observed for these secondary endpoints in the cross-sectional comparisons.

### Exploratory Analyses

**Figure 4:**
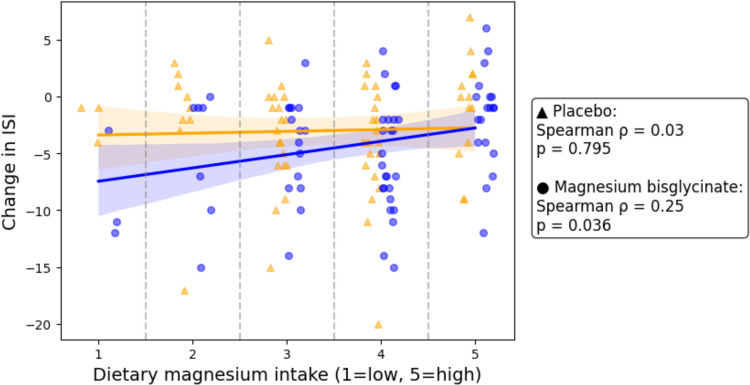
Correlation of dietary magnesium intake with ISI score changes. Scatter plot showing the correlation between self-reported dietary magnesium intake (1 = low, 5 = high) and changes in ISI scores. Results are presented separately for the placebo group (▲) and the magnesium bisglycinate group (●). Spearman correlation coefficients (*ρ*) and *p*-values are provided for each group. Shaded areas represent 95% confidence intervals.

Before the intervention, dietary magnesium intake of all participants was assessed using a non-validated single question Likert scale. A small but statistically significant inverse correlation (Spearman’s rho = −0.25, p = 0.036) was observed between magnesium intake and ISI change scores in the magnesium bisglycinate group (Figure 4). No significant correlations were observed in the placebo (p = 0.795) group.

### Sleep Diary Outcomes

As sleep diary completion was voluntary, fewer than 10% of participants provided complete entries. This limited the available data to such an extent that meaningful interpretation of sleep diary outcomes was not possible.

### Adverse Events

Reports about adverse events were collected during the study and tolerability of capsule intake was assessed at the end of the study in the questionnaires. The frequency of self-reported adverse events is detailed in Table 5. There were no reports of any serious adverse events in the trial, although two participants in the placebo group withdrew due to reported gastrointestinal issues. Most participants, specifically 93%, did not report any adverse events.

**Table 5:** Self-Reported Adverse Events in the Placebo and Magnesium bisglycinate Groups

	Placebo n = 76	Magnesium bisglycinate n = 77
**Gastrointestinal issues**	3	
**Sleep disturbances**	1	
**Headaches**	1	
**Dizziness & Nausea**		1
**Frequent urination**	1	
**Increased thirst at night**	1	
**Joint pain**		1
**Total number of adverse events**	7	2

**Notes**: The table presents the frequency of adverse events reported by participants in each group. No statistically significant differences were observed between groups, and no participants discontinued the trial due to adverse events.

## Discussion

### Summary of Findings

This nationwide, home-based, four-week, randomized, double-blind, placebo-controlled trial provides valuable insights into the effects of magnesium bisglycinate on sleep quality in individuals with self-reported insomnia symptoms. Generalized linear mixed model (GLMM) analysis confirmed a small but statistically significant reduction in ISI scores following supplementation with 250 mg elemental magnesium and 1523 mg glycine daily, with most improvements occurring within the first 14 days and sustained thereafter. This effect was further supported by per-protocol (PP) analysis, reinforcing the robustness of the findings. The effect size for ISI reduction was small (d = 0.2), indicating a modest but potentially meaningful treatment effect. However, it was well tolerated, with reported side effects occurring less frequently in the magnesium bisglycinate group than in the placebo group. Sleep quality (SQS) improved significantly in both groups within the first week, though the between-group difference did not reach statistical significance (p = 0.069).

### Comparison with Previous Research

A recent systematic review concluded that while observational studies suggest an association between magnesium status and sleep quality, interventional trials have yielded inconsistent results, highlighting the need for well-designed studies with larger sample sizes and longer durations.^22^ However, with two additional randomized trials published in 2024^21^,^46^ and the present study—the largest placebo-controlled trial on magnesium and sleep to date—growing evidence supports the role of magnesium supplementation in improving sleep outcomes.

There were no significant between-group differences in secondary outcomes. Although participants had higher baseline scores on secondary measures (eg, RIS, FSS, and PSS), no time × group interaction was observed. A possible reason for the lack of between-group effects on the RIS and SQS is their lower sensitivity compared to the ISI. The SQS consisted of a single question, providing less precise data than the ISI (7 items) and the RIS (10 items). In addition, the RIS was mainly designed to assess improvements after cognitive behavioral therapy for insomnia (CBT-I) and includes a question about sleep medication use, making it less applicable here. It was therefore used primarily as a screening tool at recruitment. Baseline ESS scores did not indicate daytime sleepiness, and the intervention period may have been too short to induce a measurable improvement.

The self-rated stress scores (PSS) in this study showed no significant reductions (p = 0.7, Cohen’s d = 0.04), indicating that the magnesium bisglycinate intervention had little to no measurable effect on stress within the four-week period. This lack of effect may reflect the short duration of the intervention or variability between participants. For example, significant reductions in PSS scores have only been observed after longer interventions, such as in an eight-week trial of another supplement.^47^ Participants in this study did not report notable mood disturbances at baseline, and while magnesium deficiency is associated with depression,^48^ no changes in mood-related parameters (PHQ-4, PANAS) were observed.

An exploratory analysis using a non-validated 1–5 visual analogue scale for magnesium-rich food and water intake suggested an inverse correlation with ISI improvements in the magnesium bisglycinate group (Spearman’s rank = 0.25, p = 0.036) but not in the placebo group (Spearman’s rank = 0.03, p = 0.795). However, the methodological limitations of this scale necessitate validation through dietary recalls and laboratory markers (eg, 24-h urine) to accurately assess magnesium levels and identify potential treatment responders to supplementation.

### Mechanisms of Action

These findings raise the question of how magnesium exerts its effects on sleep at a mechanistic level. Animal studies have extensively examined the role of magnesium in brain function and sleep regulation, suggesting that its sleep-enhancing properties may stem from both central nervous system (CNS) activity and peripheral muscle relaxation. For example, a trial in rats found that a magnesium-deficient diet was associated with neuronal excitability and disturbed sleep patterns.^49^ Another study conducted on mice found that magnesium concentrations vary by brain region, with the frontal cortex and basal forebrain showing the highest levels.^50^ Longer slow-wave sleep during recovery from sleep deprivation correlated with increased magnesium in the motor cortex, amygdala, midbrain, and cerebellum.

In vitro studies provide further insight into magnesium’s role in both the CNS and muscle function. Research on skinned skeletal muscle fibers has shown that magnesium regulates calcium movements between the sarcoplasmic reticulum and the myofilament space,^51^ promoting calcium uptake and reducing intracellular calcium levels during muscle relaxation. In the CNS, magnesium at physiologically relevant concentrations potentiates GABA_A_ receptor activity,^52^ thereby enhancing inhibitory neurotransmission, reducing neuronal excitability, and promoting relaxation, which may contribute to improved sleep quality.

Although magnesium is postulated to be the main contributor to the observed effects, glycine may provide complementary benefits. As an inhibitory neurotransmitter, glycine interacts with NMDA receptors and has been implicated in promoting relaxation and deeper sleep through mechanisms such as lowering core body temperature.^26^ The specific properties of the magnesium-glycine complex may offer advantages over other forms of magnesium.

Beyond these potential synergistic effects, the tissue-specific bioavailability of magnesium bisglycinate may further influence its effectiveness. While previous research suggests that different magnesium forms have similar intestinal absorption rates,^25^ there could be some minor variations in tissue-specific bioavailability: magnesium bisglycinate has been shown to increase brain magnesium levels at high doses in mice, whereas its effects on muscle magnesium levels appear to be minimal,^53^ suggesting a selective tissue uptake pattern. Evidence from individuals with impaired ionic magnesium absorption suggests that magnesium bisglycinate may be partially absorbed intact as a dipeptide,^54^ which could allow for potential differences in tissue-specific bioavailability after absorption.

### Study Strengths, Limitations and Directions for Future Research

This was a large, nationwide, placebo-controlled trial and the first to test magnesium bisglycinate for its effects on sleep, with both intention-to-treat (ITT) and per-protocol (PP) analyses confirming a statistically significant effect on ISI scores. Conducted as a home-based, online assessment, this trial collected real-world evidence on the impact of magnesium bisglycinate in a naturalistic setting, enhancing its applicability to daily life. The study utilized validated psychological questionnaires and included a diverse group of participants reflecting the German population affected by mild to moderate sleep disturbances, with variation in age, gender, and occupational status.

While magnesium bisglycinate significantly improved ISI scores, the mean score at week 4 remained in the subthreshold insomnia range,^45^ indicating that supplementation alone is unlikely to eliminate insomnia in many individuals. The validity of the sleep-related outcomes is limited by the exclusive reliance on self-reported data without objective verification. Future studies should incorporate objective sleep assessments, such as actigraphy or polysomnography, to corroborate self-reported improvements.

The observed exploratory inverse association between treatment response and self-reported dietary magnesium intake suggests that individuals with lower magnesium intake may benefit more from supplementation, supporting the generalizability of these findings. However, the use of an unvalidated questionnaire to assess dietary intake and the absence of biochemical markers of magnesium status raise the possibility that factors such as overall dietary quality or health status may have influenced the results. Future trials should consider using validated dietary assessment methods such as food frequency questionnaires (FFQs). They should also include biochemical markers of magnesium status, such as serum magnesium or, preferably, 24-hour urinary magnesium excretion, which may better reflect magnesium balance before and after supplementation. In addition, mechanistic studies investigating neurotransmitter activity and hormone levels (eg, cortisol) could help clarify how magnesium influences sleep regulation.

Given the complexity of magnesium’s transport and distribution, further research should investigate whether and how different magnesium formulations influence tissue-specific uptake, particularly in the brain. Future research should compare different magnesium formulations, such as magnesium citrate, taurate, and oxide, to determine if specific forms offer advantages for sleep and neurological function.

Although stress and mood (PSS, PHQ-4) were assessed, magnesium bisglycinate showed no consistent effects in this study. As these outcomes were not the focus of the intervention, future research should investigate potential psychological benefits in more specifically targeted populations.

## Conclusion

This study is the first to evaluate the effects of magnesium bisglycinate on sleep quality in a national, home-based trial with broad inclusion criteria, providing insight into its real-world applicability. The results suggest that 28 days of supplementation (250 mg elemental magnesium, 1523 mg glycine) resulted in modest but statistically significant improvements in ISI scores (d = 0.2) in adults with self-reported primary insomnia symptoms, as confirmed by both ITT and PP analyses. While no significant effects were observed in other parameters, these findings support magnesium bisglycinate as a potential non-pharmacological option for mild insomnia.

Future studies should confirm these findings in diverse populations, examine long-term effects and safety, and include objective sleep assessments. In addition, research should examine how different magnesium formulations, dosages, and baseline magnesium levels affect sleep outcomes to refine personalized recommendations.

## Data Availability

The data supporting the results and analyses presented in this study will be made available upon reasonable request in accordance with the Taylor & Francis Share Upon Reasonable Request Data Policy (https://authorservices.taylorandfrancis.com/data-sharing-policies/share-upon-reasonable-request/). Researchers interested in accessing the data should contact the corresponding author for further details. Any data sharing will be subject to ethical and privacy considerations.

## AI Assistance

During the preparation of this work, the authors used DeepL and ChatGPT (versions 3 and 4o) to assist with language phrasing and translation accuracy. All content was reviewed and edited by the authors to ensure accuracy and originality, and the authors take full responsibility for the final version of the manuscript.
